# Malaria surveillance, outbreak investigation, response and its determinant factors in Waghemra Zone, Northeast Ethiopia: unmatched case–control study

**DOI:** 10.1038/s41598-023-36918-3

**Published:** 2023-06-19

**Authors:** Habtu Debash, Marye Nigatie, Habtye Bisetegn, Daniel Getacher Feleke, Gebru Tesfaw, Askale Amha, Megbaru Alemu Abate, Alemu Gedefie

**Affiliations:** 1 Department of Medical Laboratory Sciences, College of Medicine and Health Sciences, Wollo University, Dessie, Ethiopia; 2 Department of Internal Medicine, School of Medicine, Wollo University, Dessie, Ethiopia; 3 Department of Medical Laboratory Sciences, College of Health Sciences, Woldia University, Woldia, Ethiopia; 4 Department of Microbiology, Immunology and Parasitology, College of Health Sciences, Addis Ababa University, Addis Ababa, Ethiopia; 5 Waghemra Zone Health Department, Sekota, Ethiopia; 6 Department of Medical Laboratory Sciences, College of Medicine and Health Sciences, Bahirdar University, Bahirdar, Ethiopia; 7 The University of Queensland, School of Public Health, Brisbane, Australia

**Keywords:** Microbiology, Health care, Pathogenesis, Risk factors

## Abstract

Malaria is a major global public health concern, with around half of the world's population at risk of infection. It is one of the most common epidemic-prone diseases, resulting in on-going epidemics and significant public health problems. On September 12, 2022, Waghemra Zone malaria monitoring data revealed that the district was suffering an unusually high number of malaria cases. Therefore, the aim of this study was to assess the occurrence of malaria outbreaks and investigate contracting factors in Waghemra Zone, Northeast Ethiopia. A community-based case–control study with a 1:1 ratio was employed at Waghemra Zone from September 14 to November 27, 2022. A total of 260 individuals (130 cases and 130 controls) were included in the study. A structured questionnaire was used to collect the data. Malaria cases were confirmed by either microscopy or malaria rapid diagnostic tests. The magnitude of the outbreak was described by place, person, and time. A multivariable logistic regression analysis was conducted to identify malaria risk factors. A total of 13,136 confirmed cases of malaria were detected in the Waghemra zone, with an overall attack rate of 26.5 per 1000 and slide positivity rate was 43.0%. The predominant species was *Plasmodium falciparum* accounting for 66.1%. Children under five years old (AOR = 5.1; 95% CI 2.6–23.0), the presence of artificial water-holding bodies (AOR: 2.7; 95% CI 1.340–5.420), intermittent rivers closer to the living house (AOR = 4.9; 95% CI 2.51–9.62), sleeping outside a home (AOR = 4.9; 95% CI 2.51–9.62), and a lack of knowledge about malaria transmission and prevention (AOR: 9.7; 95% CI 4.459–20.930) were factors associated with malaria contraction. The overall attack rate for malaria during this outbreak was high. Children less than five years, the presence of mosquito breeding sites, staying outdoors overnight, and a lack of knowledge on malaria transmission and prevention were predictors of malaria. Early management of local vector breeding places, as well as adequate health education on malaria transmission and prevention methods, should be provided to the community to prevent such outbreaks in the future.

## Introduction

Malaria is a widespread and debilitating tropical disease caused by *Plasmodium* species and transmitted through the bites of infected female Anopheles mosquitoes^1^. According to the World Health Organization's (WHO) 2021 malaria report, the WHO African regions continue to suffer the greatest burden of malaria. The African Region accounted for 95% of all malaria cases (228 million) and 96% of all malaria deaths (602 000) in 2020, with children under the age of five accounting for 80% of all malaria deaths in the region. Malaria services were hampered beginning in 2020 because of the Covid-19 epidemic, adding to the region's malaria load^2^.

Malaria is a major public health issue in Ethiopia, where it is estimated that 68% of the population resides^3^. Despite widespread deployment of malaria prevention strategies such as early diagnosis and treatment, indoor residual spraying, and mass distribution campaigns of long-lasting insecticide-treated bed nets^4^, Ethiopia has the highest incidence of malaria cases. Malaria is mostly an endemic disease in the country, and outbreaks sometimes happen. Its transmissions peak between September and December, following the main rainy season, and between June and August^3^.

Recurrent outbreaks and epidemics are linked to cyclical weather fluctuations in the country, which lead to enhanced vector survival. Other triggering factors include exceptional local weather events and activities that result in environmental alteration, increasing vector populations, and increasing population vulnerability to famine, starvation, and conflict^3,5^. More than 542,000 people have been displaced as a result of internal conflict in Amhara region Ethiopia. The Waghemra zone has been severely affected by this internal conflict^6^. The conflict has led to the deterioration of health services, the interruption of anti-malarial treatments, and the movement of people, which has resulted in the failure of efforts to keep malaria under control and the likelihood of an outbreak^7^.

The Waghemra zone is one of the most malaria-prevalent areas in the Amhara region of northeast Ethiopia. On September 12, 2022, malaria monitoring data obtained from the Zone Health Office revealed that the districts were experiencing an exceptionally high number of malaria cases. In WHO epidemiologic week 36 of 2022, a total of 190 malaria cases were registered, compared to only 122 cases in the same epidemiologic week during the threshold period (2016–2020). On September 14, 2022, a rapid response team was dispatched to the affected districts to confirm the existence of the outbreak, identify risk factors, and aid in intervention actions.

Understanding the causes of outbreaks in these areas allows for early case management, identification of variables that maintain the disease, and the design of more effective preventative and control methods to facilitate malaria elimination by 2030. As a result, the goal of this study was to confirm the occurrence of the outbreak, identify gaps and risk factors that contributed to the outbreak's existence, and provide appropriate public health intervention for the outbreak in the Waghemra zone.

## Materials and methods

### Study area

Waghemra Zone is one of eleven zones in Amhara region of Ethiopia. The Waghemra zone is defined by the following latitude and longitude coordinates: 12° 45′ 54" N, 38° 50′ 34.8"E and has an elevation of 1498 m. In terms of health care, it has 136 health posts, 34 health centers, one general hospital, and two primary hospitals. This zone is divided into eight districts with a total population of 536,129 people. Data was collected from Ziquala, Sahala, Abergelie, Dehana, Sekota Zuria, Sekota Town and Gazgibla districts. However, due to the presence of war during data collection in the Tsagbji district and some kebeles in the Abergele district were excluded. The outbreak occurs in all districts, but the severity varies. The area's average yearly temperature and rainfall are 26 °C and 786 mm, respectively. The climate and topography of the study areas are conducive to Anopheles mosquito breeding, and malaria transmission is prevalent.

### Study design and period

Community based unmatched case–control study was conducted from September 14 to November 27, 2022.

### Source population, study subject and variables

People living in the Amhara region's Waghemra zone who are at risk of malaria are the source population. And the specific study subjects for these cases were febrile patients who tested positive for malaria parasites by either Rapid Diagnostic test (RDT) or a microscope. Controls, on the other hand, were classified as having no signs and symptoms of acute febrile illness one month before data collection. A non-febrile, apparently healthy person living in the same village as the active case patient from September 14 to November 27, 2022, was studied as a control subject. Controls were selected regardless of their age, gender, educational status, physiological status, and socio-economic status. The independent variables were socio-demographic and economic characteristics, behavioral factors like Insecticide-Treated Nets (ITN) use, Indoor Residual Spray (IRS), sleeping area at night and environmental factors.

## Descriptive and analytical epidemiology

### Confirm the diagnosis and verify the existence of the outbreak

Malaria data from the last six years (2016–2021) were analyzed at the Waghemra zone health office to determine the epidemic threshold level. However, because of the inadequacy of the most recent year's (2021) data, the previous five years' (2016–2020) weekly malaria case reports were utilized. Then epidemic threshold level was defined by comparing weekly data with similar weeks in 2022, and an epidemic curve was produced. A rise beyond the weekly threshold was recorded, indicating an outbreak. On September 12, 2022 (week 36), an early warning alarm was received from the Waghemra zone. The Zonal public health emergency management case team decided to investigate or confirm the outbreak and intervene after receiving a request from the zone health office and analyzing regular surveillance data. A number of malaria cases have been recorded; the slide positivity rate and attack rate were calculated as the number of confirmed malaria cases per 100 and 1000 population, respectively.

### Sample size determination and sampling technique

The sample size was calculated using Epi-Info version 7.2.1 by taking an 80% power,, an odds ratio of 3.32 for the presence of artificial water holding bodies near the home, the percentage of exposed controls of 21.3%^8^, and the case-to-control ratio of 1:1. The total sample size was 118. Considering a design effect of 2 and 10% non-response rate, the final sample size became 260, with 130 cases and 130 controls**.**

A multi-stage random sampling method was used to enrol the study participants. Waghemra zone has eight districts, and of them, three (Ziquala, Sahala, and Abergelie) were purposefully selected. In each district, two kebeles were selected randomly using a lottery method. Accordingly, Tsitsika and Netsawork, Silazge and Meharit, and Saka and Debre-brihan kebeles were selected from Ziquala, Sahala, and Abergele districts, respectively. The total households for each village were available at their nearest health center or health post, which is stored as a family card folder. Based on this, the total sample size was proportionally allocated as 60, 43, 52, 33, 47, and 25 to Tsitsika, Netsawork, Silazge, Meharit, Saka, and Debre-brihan kebeles, respectively. All cases and controls were selected from the same community or neighbour for the controls at the same time. The lottery method was applied to select individual participants in the selected household.

### Data collection

Six health extension workers and six laboratory technologists collected data using a structured questionnaire under the supervision of the principal investigator and the zonal public health emergency management case team. The questionnaire utilized in the study was prepared by reviewing the literatures^7–9^. Data collectors and supervisors received one day of training to ensure data quality. A review of weekly Integrated Disease Surveillance and Response (IDSR) reports at various levels (district health office and health facilities) was done. For adults, selected cases and controls were interviewed directly; for children, parents were involved in the interview process. But each participant gave blood for malaria diagnosis.

### Laboratory methods

At Waghemra Zone health facilities, laboratory technologists utilized a light microscope to detect malaria parasites. During power outages, RDTs were used in healthcare facilities. Furthermore, at time of outbreak investigation, health extension workers and surveillance teams employed RDTs to identify confirmed malaria cases at health posts and the community level.

### Environmental and vector control assessment

The environmental impact, as well as the ownership and use of ITNs were assessed. Selected case patients and controls were asked questions regarding the existence of mosquito breeding places in and around their compound. The potential breeding sites of Anopheles mosquitoes, such as uncovered plastic water containers, old tires, stagnant water, and broken glasses in the home or outside the home were evaluated. Furthermore, we assessed for the presence of anopheles’ larvae in stagnant water.

### Data processing and analysis

Data were entered into Epi-Info 7.2.0.1 and analyzed using Statistical Package for Social Science version 26 (SPSS-26). The outbreak's scope was described in terms of person, place and time. The significance of risk factors for the outbreak was determined using logistic regression. Variables with p-value < 0.25 in bivariate analysis were entered in multiple logistic regression analysis to examine the effect of an independent variables on the outcome variable. The association between dependent and independent variables was determined using Odds Ratio (OR) of 95% Confidence Interval (CI) at p-value less than 0.05 was regarded as statistically significant.

### Ethical consideration

Ethical clearance was obtained from the ethical review committee of College of Medicine and Health Sciences, Wollo University on the date 16/8/2022 with a protocol number of CMHS/201/2022. Supportive letters were also obtained from the Waghemra Zone Health Office. Written informed consent and assent were obtained from participants or caregivers. Positive cases were treated according to national malaria guidelines. The information obtained was made anonymous and de-identified prior to analysis to ensure confidentiality. The study was also conducted in accordance with the Helsinki Declaration.

## Results

### Socio demographic characteristics

During the study period, 260 eligible study participants were selected and interviewed, making the response rate 100. The study included 155(59.6%) males and 105 (40.4%) females. The majority of the participants were between the ages of 15 and 45. In terms of occupation and education, 124 (47.7%) were farmers, while 227 (68.8%) were illiterate (Table 1).

**Table 1:** Socio-demographic characteristics of study participants of malaria outbreak, Waghemra Zone, Northeast Ethiopia, 2022.

Characteristics	Alternatives	Case (n = 130) No (%)	Controls (n = 130) No (%)	Total (n = 260) No (%)
Sex	Male	81 (62.3)	74 (56.9)	155 (59.6)
	Female	49 (37.7)	56 (43.1)	105 (40.4)
Age group (years)	< 5	24 (18.5)	8 (6.2)	32 (12.3)
	5–14	36 (27.7)	24 (18.5)	60 (23.1)
	15–45	49 (37.7)	68 (52.3)	117 (45.0)
	> 45	21 (16.2)	30 (23.1)	51 (19.6)
Occupation	Government employee	5 (3.8)	8 (6.2)	13 (5.0)
	Unemployed	3 (2.3)	7 (5.4)	10 (3.8)
	Pastoralist	41 (31.5)	34 (26.2)	75 (28.8)
	Student	14 (10.8)	24 (18.5)	38 (14.6)
	Farmer	67 (51.5)	57 (43.8)	124 (47.7)
Marital status	Married	58 (44.6)	56 (43.1)	114 (43.8)
	Single	66 (50.8)	73 (56.2)	139 (53.5)
	Others	6 (4.6)	1 (0.8)	7 (2.7)
Educational status	Illiterate	91 (70.0)	82 (63.1)	173 (66.5)
	Primary	26 (20.0)	28 (21.5)	54 (20.8)
	Secondary and above	13 (10.0)	20 (15.4)	33 (12.7)

## Descriptive result

### Description of cases by person and place

During the outbreak investigation period from WHO weeks 29 to 47, a total of 13,136 confirmed cases of malaria from the Waghemra zone were detected. Total slide positivity rate (TPR) and attack rate (AR) were 43.0% and 26.5%, respectively. From all malaria confirmed cases, the most affected age group was > 15 years (65.6%), followed by 5–14 years (24.0%), and below 5 years (10.4%). The districts with the largest proportions of malaria-confirmed patients were Ziquala, Sahala, and Abergele, with 37.9%, 37.2%, and 10.2%, respectively. On the other hand, the highest attack rate was observed in the Sahala, Ziquala, and Abergele districts, with rates of 172.2, 113.2, and 28.9, respectively. *Plasmodium falciparum* responsible for 8681 (66.1%) of the infections, while *P. vivax* responsible for 3875 (29.5%) (Table 2).

**Table 2:** Distribution of malaria cases by cluster, age group and attack rate in Waghemra Zone, Northeast Ethiopia, 2022.

Name of Woreda	Total population	Microscopy/RDT done	Confirmed malaria	PF	PV	Mixed (PF + PV)	< 5 years	5–14 years	> 15 years	TPR%	AR per 1000
Ziquala	44,013	10,073	4981 (37.9)	3588 (72.0)	1107 (22.2)	286 (5.7)	443 (8.9)	1081 (21.7)	3457 (69.4)	49.4	113.2
Sahala	28,404	11,712	4892 (37.2)	3235 (66.1)	1456 (29.8)	201 (4.1)	526 (10.8)	1351 (27.6)	3015 (61.6)	41.8	172.2
Abergelie	46,509	2118	1345 (10.2)	1084 (80.6)	237 (17.6)	24 (1.8)	195 (14.5)	268 (19.9)	882 (65.6)	63.5	28.9
Dehana	142,043	1974	616 (4.7)	236 (38.3)	362(58.8)	18 (2.9)	15 (2.4)	116 (18.8)	485 (78.7)	31.2	4.3
Sekota Zuria	100,828	2848	843 (6.4)	412 (48.9)	397 (47.1)	34 (4.0)	156 (18.5)	249 (29.5)	438 (52.0)	29.6	8.4
Sekota Town	46,041	1335	324 (2.5)	83 (25.6)	232 (71.6)	9 (2.8)	17 (5.2)	59 (18.2)	248 (76.5)	24.3	7.0
Gazgibla	87,052	512	135 (1.0)	43 (31.9)	84 (62.2)	8 (5.9)	11 (8.1)	28 (20.7)	96 (71.1)	26.4	1.6
Overall total	494,890	30,572	13,136	8681 (66.1)	3875 (29.5)	580 (4.4)	1363 (10.4)	3152(24.0)	8621(65.6)	43.0	26.5

PF: Plasmodium falciparum; PV: Plasmodium vivax.

### Description of cases by time

**Figure 1:**
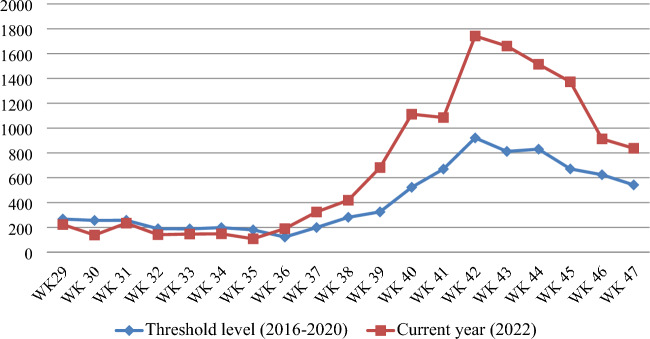
Malaria outbreak line graph by WHO epidemiologic week in Waghemra zone, Northeast Ethiopia, 2022.

The Waghemra Zone Health Department was informed that the number of malaria cases had exceeded the threshold in the WHO epidemiologic week 36/2022. The number of malaria patients steadily increased and peaked in week 42. Then it steadily decreased from week 43 to week 47 but was not controlled till this investigation was completed (Fig. 1). The intervention began with mass diagnosis using RDT and microscopy, and the positive cases were treated with artemisinin-based combination therapy and chloroquine for infection with *P. falciparum* and *P. vivax*, respectively. Health education, environmental management, distribution of ITN and the use of Abet chemicals to larvicide stagnant water were also applied.

## Analytic results

### Factors associated with malaria outbreaks

In a multivariable analysis, children under the age of five were five times more likely than those over the age of 45 to contract malaria (Adjusted Odds ratio (AOR) = 5.1; 95% Confidence Interval (CI) 2.6–23.0). People who were living in households where artificial water-holding bodies were thus 2.7 times more at risk of getting malaria infection than their counterparts (AOR: 2.7; 95% CI 1.340–5.420). Similarly, the presence of intermittent rivers closes to the community within 1 km distance increased the likelihood of getting malaria than those far away from it (AOR: 9.4; 95% CI 4.8–18.0). Likewise, children who stayed outside at night had an almost five-fold greater risk of acquiring malaria compared to those who did not (AOR = 4.9; 95% CI 2.51–9.62). Furthermore, higher odds of malaria were noted among those who had no knowledge on malaria transmission, prevention and control mechanisms (AOR: 9.7; 95% CI 4.459–20.930) (Table 3).

**Table 3:** Bivariable and multivariable analysis of risk factors for malaria outbreak in Waghemra Zone, Northeast Ethiopia, 2022.

Factors	Category	Cases = 130	Controls = 130	COR (95% CI)	*P* value	AOR (95% CI)	P-value
		No (%)	No (%)				
Age group (years)	< 5	24 (18.5)	8 (6.2)	4.286(1.616–11.366)	0.003*	5.109(2.619–23.084)	< 0.001**
	5–14	36 (27.7)	24 (18.5)	4.163(1.726–10.041)	0.001*	3.270(1.392–14.386)	< 0.001**
	15–45	49 (37.7)	68 (52.3)	2.000(0.772–5.184)	0.154*	2.409(1.210–13.654)	0.003**
	> 45	21 (16.2)	30 (23.1)	1		1	
Sex	Male	81 (62.3)	74 (56.9)	1.251(0.762–2.055)	0.377		
	Female	49 (37.7)	56 (43.1)	1			
Travel history	Yes	32 (24.6)	8 (6.2)	4.980(2.195–11.296)	0.001*	2.531 (0.864–7.413)	0.090
	No	98 (75.4)	122 (93.8)	1			
Presence of artificial water holding bodies near to home	Yes	76 (58.5)	42 (32.3)	2.949(1.777–4.895)	< 001	2.695(1.340–5.420)	0.005**
	No	54 (41.5)	88 (67.3)				
Presence of intermittent rivers with 1 km radius	Yes	86 (66.2)	29 (22.3)	6.807(3.928–11.798)	0.001*	13.844(6.180–31.009)	0.001**
	No	44 (33.8)	101 (77.7)	1			
Outdoor stay over night	Yes	63 (48.5)	19 (14.6)	5.493(3.027–9.969)	< 0.001*	6.323(2.878–13.896)	< 0.001**
	No	67 (51.5)	111 (85.4)	1			
Utilization of functional ITN	Yes	68 (52.3)	72 (55.4)	1			
	No	62 (47.7)	58 (44.6)	1.132 (0. 695–1.844)	0.619		
Number of functional ITN per household	Adequate	56 (43.1)	66 (50.8)	1			
	Inadequate	74 (56.9)	64 (49.2)	1.363(0.836–2.221)	0.214*	0.914(0.431–1.940)	0.815
Environmental control	Yes	19 (14.6)	39 (30.0)	1			
	No	111 (85.4)	91 (70.0)	2.504(1.354–4.628)	0.003*	2.098(0.877–5.017)	0.096
Knowledge on malaria transmission and prevention	Good	40 (30.8)	97 (74.6)	1			
	Poor	90 (69.2)	33 (25.4)	6.614(3.843–11.382)	0.001*	9.661(4.459–20.930)	< 0.001**

*Significant variable in bivariate analysis ** significant variables in multivariable analysis.

## Public health interventions

### Early diagnosis and treatment

During the investigation period, an active case detection was conducted using RDT or microscopy, as well as early case management in accordance with national malaria treatment standards^9^. Temporary diagnosis and treatment sites were established to control and prevent further transmission through early treatment.

### Environmental assessment

There were many mosquito breeding sites detected in the districts, which could be the source of the outbreak. In most of houses, unnecessary weeds, fake water-holding containers, especially damaged gutters, unused cans, unused old ties and stagnant waters were observed. Environmental management such as filling, draining, and clearing were carried out in an area larger than 432,157 square meters in a selected Anopheles mosquito breeding site. The community was involved in both the opening of temporarily stagnant water and the administration of larvicide (abet insecticide) at the breeding location. In this environmental management a total of 8,654 people were participated.

### Vector control activities

The zone fast response team assessed and provided vector control activities in the study area. In all households in the Waghemira zone, indoor residual spray chemicals were not sprayed due to conflict in the last year. The fast response team, sprayed anti-larval chemical (abate) on stagnant water with an approximate area of 432,157 square meters. Fifty homes from each affected kebeles were randomly selected and visited to look for new malaria cases and assess the use of insecticide-treated bed nets at night. Even though every household had at least one insecticide-treated bed net, only 42.6% of them hung it directly on the bedding, with the rest hanging it underneath the beds and elsewhere in the house Moreover, about 22.6% of the household nets were damaged. The response teams then distributed over 3100 ITNs to the community.

### Health education and communication

Health professionals were mobilized and assigned to the affected village for an active case search and early case management in the community. In addition, health education was given to 15,890 people about the cause, transmission, prevention, and control of malaria. Communicating and discussing the trend of the malaria situation with health facilities, Woreda, and zone health departments, and there was also multi-sectorial integration for social mobilization and prevention of malaria.

## Discussion

Based on five years of epidemiological records of malaria cases, the study findings showed the presence of a malaria outbreak in the study area. The malaria outbreak investigation included WHO weeks 36 to 47. Overall, the outbreak decreased but was not controlled due to inadequate environmental and vector control interventions in affected areas. For the past year, there has been an internal conflict in the study area, which has resulted in the deterioration of the health system and the interruption of malarial prevention measures, which have kept malaria under control.

The national malaria prevention and control strategies recommend the application of the IRS at least once a year with 100% coverage and at least one ITN per two people in high malaria-risk areas^10^. Despite this fact, prior to the outbreak, IRS was not applied, early replacement of ITN was not done, and there were multiple mosquito breeding sites. Households that had been using the ITN for purposes other than their intended purpose were also observed. This could be due to poor monitoring of the communities after distributing the ITN. The districts were also inadequately prepared for the outbreak, leading to a shortage of resources. This negatively affected outbreak control and resulted in the outbreak taking longer to contain. A similar finding was reported in Binga district, Zimbabwe^11^.

The overall attack rate (AR) was 26.5 cases per 1000 population; this finding was higher than a study done in Argoba district, South Wello Zone (AR: 1.8)^12^, Laelay Adyabo district, Northern Ethiopia (AR: 12.1)^13^, and India (AR: 15.1)^14^. However, this finding was lower than a study done in the Abergelle district, North Ethiopia (AR: 33.1)^15^, Simada district, Northwest Ethiopia (AR: 200)^8^, Afar region, Ethiopia (AR: 36.7)^16^, Bolosso Sore district, Southern Ethiopia (AR: 36.4)^17^, BenaTsemay district, Southern Ethiopia (AR: 114)^18^, and Kole district, Uganda (AR = 68)^19^. This difference might be attributed to prevention and control efforts, community level of awareness, internal conflict, and area differences in the burden of malaria and duration of the disease.

The AR was highest in Sahala, Ziquala, and Abergele districts, with rates of 172.2, 113.2, and 28.9 per 1,000 populations, respectively. This might be due to the presence of multiple mosquito breeding sites near residents of these districts compared to the other districts. Moreover, these districts are extremely hot and low-land areas with a high malaria burden. This was in line with a study done in the Metema district and in the Amhara Regional State, Ethiopia^20,21^. This could be due to high temperatures in the area, which are conducive to mosquito development rates, biting rates, and parasite survival within the mosquito^22^.

The greatest number of malaria cases was found in patients above the age of 15 (8621 out of 13,136). This finding was in line with studies from Abergele district Northeast Ethiopia^23^, Ankasha district, North Ethiopia^9^, and BenaTsemay district, Southern Ethiopia^18^. This might be due to the fact that the majority of the adolescents were spending more time outdoors in this area for farming, livestock-keeping, and fishing activities that exposed them to mosquito bites. This implies that the regional health bureau needs to give more focus and extend medical services to people who are engaged in farming, livestock keeping, and fishing.

The predominant *Plasmodium* species detected in this study was *P. falciparum* (66.1%), followed by *P. vivax* (29.5%). This was in agreement with other previous studies done in Argoba district, Northeast Ethiopia^12^, and Abergele district, Northern Ethiopia^15^. However, it disagreed with the national malaria parasite distribution pattern of Ethiopia, which showed that *P. falciparum* and *P. vivax* accounted for 60 and 40% of the malaria cases in the country, respectively^24^. This variation could be due to the fact that this study was limited to a small malaria-endemic setting in the country, which could have caused the species prevalence to vary. In addition, *P. falciparum* is a common species in the lowlands.

Malaria outbreaks are frequently complicated and multi-factorial, including both natural and man-made causes^25^. This case–control study verified the occurrence of a malaria outbreak in the Waghemra zone. Age, the availability of artificial water-holding bodies, nearby stagnant water, sleeping outside overnight, and a lack of knowledge about malaria transmission and prevention all contributed to the epidemic's existence. As a result, children under the age of five were nearly five times more likely than individuals over the age of 45 to contract malaria. This finding was congruent with research undertaken in the Bena Tsemay district of southern Ethiopia^18^. Malaria immunity develops slowly after multiple infections, and it takes at least five years for children to establish immunity^26^.

Furthermore, people who live near artificial water-holding bodies and stagnant water were more likely to be exposed to the malaria parasite than their counterparts. A similar conclusion was reached in research conducted in Simada district, Northwest Ethiopia, which found a link between staying near such water sources and contracting malaria^8^. Stagnant water created by heavy rains provides an ideal breeding environment for mosquitoes and contributes to malaria epidemics^8,16^. Similarly, people who stayed outside at night were approximately five times more likely to be infected with malaria than those who did not. This finding was supported by a report from the Ziquala, Armachiho, and Dembia districts of the Amhara region in Ethiopia^27–29^. This could be explained by the exophagic-exophilic biting behaviours of mosquitoes^30^. Moreover, a lack of knowledge regarding malaria transmission and control was a risk factor for disease development. Malaria education is crucial for minimizing exposure to the disease and its negative health consequences^8,31,32^.

During the investigation period, active case searching, treatment and management were carried out in accordance with national malaria treatment guidelines. Aside from that, environmental management activities such as filing, draining and clearing temporarily stagnant water were done with community involvement. At the time of data collection period, larvicide (abet chemical) was sprayed on Anopheles mosquito breeding sites. Moreover, the malaria surveillance team provided health education on disease transmission and prevention, and distributed over 3100 ITN to the community. However, due to a scarcity of chemicals, indoor residual spraying of houses in impacted kebeles is now being delayed. This outbreak scenario exemplified the critical role of long-term environmental and vector control intervention through well-organized malaria strategies and programs in preventing and controlling malaria infections. Malaria control and elimination require cross-sectoral collaboration as well as close monitoring and assessment of prevention and control initiatives.

## Conclusion and recommendations

Following a year of internal conflict, a malaria outbreak was confirmed in Waghemra Zone. The predominant *Plasmodium* species identified was *P. falciparum*, and the outbreak was linked to being under five age, the existence of vector-breeding areas, people staying outdoors overnight, and a lack of knowledge about malaria transmission and control. The response to the outbreak included early diagnosis and treatment, environmental change, vector control, and awareness raising, which resulted in a reduction but not complete control of the outbreak. To prevent future malaria outbreaks in the study area, we recommended that the Waghemira Zone health office, Amhara regional health bureau, and other concerned sectors implement the following malaria prevention and control techniques: Those include raising community knowledge about malaria, mobilizing to disrupt mosquito breeding areas, scheduling indoor residual spraying activities, and monitoring malaria case trends on a weekly basis.

### Ethical approval and consent to participate

Ethical clearance was obtained from the ethical review committee of College of Medicine and Health Sciences, Wollo University on the date 16/8/2022 with a protocol number of CMHS/201/2022. Permission was obtained from Waghemra Zone Health Office and each district health office where the study was conducted. This study was conducted in accordance with the Declaration of Helsinki. After briefly describing the significance of the study, the participants or children’s parents or guardians signed informed written consent. Confidentiality of the data was maintained. Finally, participants who were infected with the *Plasmodium* parasite received antimalarial treatment according to the national malaria treatment guidelines.

## Data Availability

All relevant data are included in the published article.

## Abbreviations

OR: Odds ratio
AR: Attack rate
CI: Confidence interval
IRS: Indoor residual spray
ITN: Insecticide-treated nets
PF: *Plasmodium falciparum*
PV: *Plasmodium vivax*
RDT: Rapid diagnostic test
SPSS: Statistical Package for Social Sciences
TPR: Total slide positivity rate
WHO: World Health Organization
